# Supplementary material for: Towards equitable scheduling of global health teleconferences: a spatial exploration of the world’s population and health by time zone
Source: BMJ Open. 2022 May 30;12(5):e056696. doi: 10.1136/bmjopen-2021-056696 (PMC9152942; doi:10.1136/bmjopen-2021-056696)
Supplement: Supplementary data [file bmjopen-2021-056696supp001.pdf]

Supplementary Figure. Static image of interactive three-dimensional UTC split-plot of global population, countries, human development index, total and disease-specific disability adjusted life years per 100,000 by simplified Coordinated Universal Time offset, 2019-20

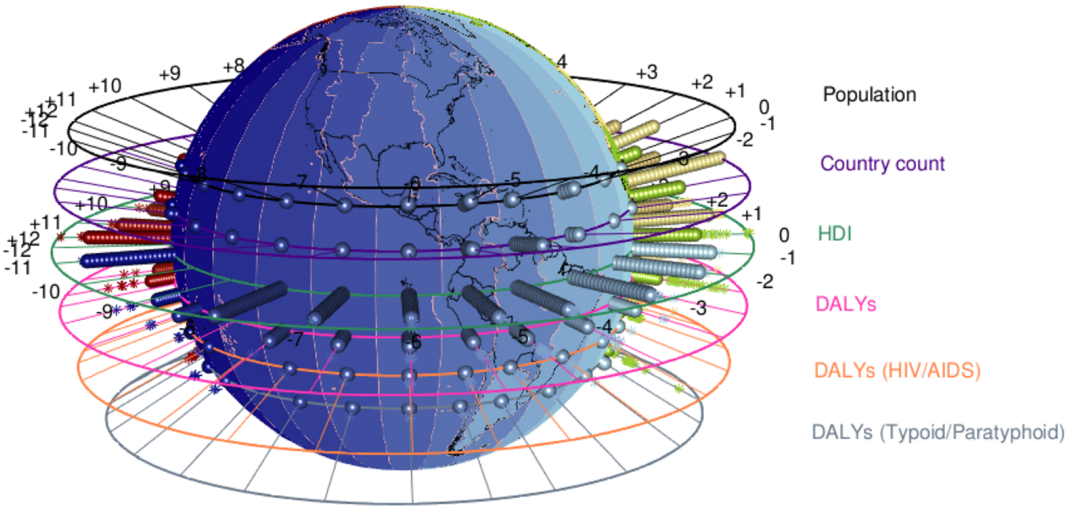

HDI: Human development index; DALYs: disability-adjusted life years; UTC: Coordinated Universal Time.
Interactive version available at:
https://www.stats.otago.ac.nz/~tdavies/utcsplit_health.html
(allow up to 30 seconds to load in browser)
