# Supplementary material for: Towards equitable scheduling of global health teleconferences: a spatial exploration of the world’s population and health by time zone
Source: BMJ Open. 2022 May 30;12(5):e056696. doi: 10.1136/bmjopen-2021-056696 (PMC9152942; doi:10.1136/bmjopen-2021-056696)
Supplement: Author's manuscript [file bmjopen-2021-056696.draft_revisions.pdf]

BMJ Open

BMJ Open is committed to open peer review. As part of this commitment we make the peer review history of every article we publish publicly available.

When an article is published we post the peer reviewers' comments and the authors' responses online. We also post the versions of the paper that were used during peer review. These are the versions that the peer review comments apply to.

The versions of the paper that follow are the versions that were submitted during the peer review process. They are not the versions of record or the final published versions. They should not be cited or distributed as the published version of this manuscript.

BMJ Open is an open access journal and the full, final, typeset and author-corrected version of record of the manuscript is available on our site with no access controls, subscription charges or pay-per-view fees (http://bmjopen.bmj.com).

If you have any questions on BMJ Open's open peer review process please email info.bmjopen@bmj.com

BMJ Open

Towards equitable scheduling of global health teleconferences: a spatial exploration of the world's population and health by time zone

Journal:	*BMJ Open*
Manuscript ID	bmjopen-2021-056696
Article Type:	Original research
Date Submitted by the Author:	22-Aug-2021
Complete List of Authors:	Crump, John; University of Otago, Centre for International Health<br>Davies, Tilman ; University of Otago, Department of Mathematics and Statistics
Keywords:	MEDICAL ETHICS, Telemedicine < BIOTECHNOLOGY & BIOINFORMATICS, EPIDEMIOLOGY, International health services < HEALTH SERVICES ADMINISTRATION & MANAGEMENT, OCCUPATIONAL & INDUSTRIAL MEDICINE, STATISTICS & RESEARCH METHODS

SCHOLARONE™
Manuscripts

I, the Submitting Author has the right to grant and does grant on behalf of all authors of the Work (as defined in the below author licence), an exclusive licence and/or a non-exclusive licence for contributions from authors who are: i) UK Crown employees; ii) where BMJ has agreed a CC-BY licence shall apply, and/or iii) in accordance with the terms applicable for US Federal Government officers or employees acting as part of their official duties; on a worldwide, perpetual, irrevocable, royalty-free basis to BMJ Publishing Group Ltd ("BMJ") its licensees and where the relevant Journal is co-owned by BMJ to the co-owners of the Journal, to publish the Work in this journal and any other BMJ products and to exploit all rights, as set out in our licence.

The Submitting Author accepts and understands that any supply made under these terms is made by BMJ to the Submitting Author unless you are acting as an employee on behalf of your employer or a postgraduate student of an affiliated institution which is paying any applicable article publishing charge ("APC") for Open Access articles. Where the Submitting Author wishes to make the Work available on an Open Access basis (and intends to pay the relevant APC), the terms of reuse of such Open Access shall be governed by a Creative Commons licence – details of these licences and which Creative Commons licence will apply to this Work are set out in our licence referred to above.

Other than as permitted in any relevant BMJ Author's Self Archiving Policies, I confirm this Work has not been accepted for publication elsewhere, is not being considered for publication elsewhere and does not duplicate material already published. I confirm all authors consent to publication of this Work and authorise the granting of this licence.

For peer review only

Towards equitable scheduling of global health teleconferences: a spatial exploration of the world's population and health by time zone

John A. Crump, MB ChB, MD, DTM&H[1]

Tilman M. Davies, PhD[2]

[1]Centre for International Health, Otago Medical School, University of Otago, Dunedin, New Zealand

[2]Department of Mathematics and Statistics, University of Otago, Dunedin, New Zealand

Corresponding author: Prof. John A. Crump, Centre for International Health, Otago Medical School, University of Otago, PO Box 56, Dunedin, 9054, New Zealand. Tel +64-3-479-9460, Email: john.crump@otago.ac.nz

Word count: 2,890

For peer review only

ABSTRACT

Objectives To examine the world's population, development, and health by Coordinated Universal Time (UTC) offset and to consider the findings in the context of the ethics of distribution.

Design Spatial exploration examined through the lens of the ethics of distribution.

Setting The earth.

Participants The global population.

Main outcome measures Global population, countries, human development index, and health measured in total and disease-specific disability adjusted life years (DALYs) per 100,000 per year by UTC offset. Strengths and weaknesses of scheduling teleconferences accordingly to alternative approaches to the ethics of distribution.

Results The UTC offset world population maximum of approximately 1,724 million persons occurred in UTC+5, country count maximum of approximately 40 countries in UTC+1, and the median human development index minimum of 0.527 occurred at UTC0. The median total DALYs per 100,000 per year maximum of 41,873 occurred in UTC+11, median HIV/AIDS DALYs per 100,000 per year maximum of 941 in UTC0, and median typhoid and paratyphoid fevers DALYs per 100,000 per year maximum of 279 occurred at UTC+5. Hypothetical teleconference scheduling

For peer review only

For peer review only - http://bmjopen.bmj.com/site/about/guidelines.xhtml

scenarios generated temporal distances of up to 11 hours to UTC offsets representing the most countries and greatest number of DALYs per 100,000 per year. Teleconference scheduling based on egoism was considered to be problematic, and contractualism as unrealistically demanding. Utilitarianism resulted in the risk of systematically disadvantaging small, temporally remote groups. Egalitarianism offered equality, but prioritarianism offered the possibility of addressing health inequities.

Conclusions Global health teleconferences may generate great temporal distances between participants that can compromise representative participation, as well as the wellbeing and health of attendees. Our spatial exploration of the world's population and health by UTC offset may be used to inform prioritarian approaches to scheduling international teleconferences that may enhance progress towards the global health aspiration of health equity.

For peer review only

ARTICLE SUMMARY

Strengths and limitations of this study

- First study to display world's population and health by time zone to inform global health teleconference scheduling in the context of the ethics of distribution.
- We demonstrate that multiple time zone international teleconferences can yield large temporal distances between participants.
- Using hypothetical examples, we illustrate how egoisim and ultilitarianism may make it difficult for participants in highly affected areas to participate, or may systematically exclude participants and remote and less populous time zones.
- For ease of presentation and interpretation we chose to 'shrink' time zones representing fractions of hours towards zero resulting in conflation of the 37 time zones to UTC and its 24 offsets.

For peer review only

INTRODUCTION

Addressing global health problems through collaboration depends in part on international synchronous communication and coordination.[1] In modern times, this is typically achieved by teleconference, a collective term for communication using a range of platforms including voice only conference calls, videoconferences, and web-based meetings. Addressing the COVID-19 pandemic has at once demanded increased levels of global health communication and coordination, and driven restrictions on face-to-face meetings[2] resulting also in a marked increase in demand for international teleconferences for communication on a wide range of health conditions. While teleconferences have been considered inferior to face-to-face meetings for complex tasks with high ambiguity and where new ties need to be built,[3] the aspiration to pursue more judicious use of cleaner international air travel following the COVID-19 pandemic[4] point to a future with a sustained high level of international teleconference use.

A consequence of the earth approximating a sphere that orbits the sun and spins, daylight is experienced in sequence by longitude on a 24 hour cycle. To manage this, the world is presently organised into 37 longitudinal time zones adapted to follow national and subnational boundaries, and observed as Coordinated Universal Time (UTC) offsets from the prime meridian in Greenwich, England, the international standard for zero degrees longitude,[5] and home to UTC offset zero or 'zulu time.' Temporal distance is a measure, distinct from geographic distance, defined as a directional measurement of the temporal displacement experienced by two collaborators who want to interact with each other.[6] Because humans are a diurnal species with a circadian clock entrained closely to light, traditionally from the sun,[7]

For peer review only

the temporal distance inherent in synchronous multiple time zone teleconferencing poses a major challenge to representative participation[8] and to supporting the health and wellbeing of attendees.[9] Methods in common use for scheduling international conference calls include online tools facilitating identification of times convenient to the greatest number of potential participants, a form of utilitarianism. However, scheduling may be influenced by other drivers including egoism, where the preferences of the organiser are prioritised. In teleconference spanning most or all global time zones, some participants will be inevitably be asked to join between the hours of 11pm through 5am local time, a time window classified as unfavourable by some online teleconference scheduling tools. But who should be asked to join at these times and why? While global health claims to seek health equity,[1] it is recognized that aspects of its history in colonial medicine, missionary medicine, tropical medicine, and international health may perpetuate inequities.[10] The decolonising global health movement 'fights against ingrained systems of dominance and power in the work to improve the health of populations, whether this occurs between countries, including between previously colonising and plundered nations, and within countries.'[11] To our knowledge, there has hitherto been little specific attention given to supporting equity in the organisation and scheduling of international teleconferences in global health.

For peer review only

In order to inform equitable approaches to scheduling multiple time zone teleconferences in global health, we sought to describe empirical data on the world's population and its health by time zone. The primary objectives of this analysis were to display human population characteristics and health by time zone and to consider the implications of these findings in the context of the ethics of distribution. We

For peer review only - http://bmjopen.bmj.com/site/about/guidelines.xhtml

aspired to identify strategies to enhance equitable organisation and participation, and ultimately outcomes, for teleconferences in global health.

METHODS

We undertook a spatial exploration of the world's population and its health by time zone. We illustrated the implications of the findings with worked examples, and assessed alternative strategies for international teleconference scheduling in light of the ethics of distribution.

Time zones

We constructed a fine grid of longitude coordinates ranging from -180º to 180º and of latitude coordinates ranging from -90º to 90º and fed the grid into the lutz ('look up time zones') software[12] in the R programming environment.[13] Each coordinate was linked to a country and region identifier, and to a non-daylight savings time UTC offset value. For simplicity, fractional UTC offsets were 'shrunk' towards zero. For example, UTC+8.5 translated to UTC+8, and UTC-3.5 to UTC-3. For countries with multiple time zones, their contributing UTC values were first averaged, and then the shrinking translation was performed. Multiple time zone country data were assigned to that single shrunk time zone. A small number of countries with UTC offsets greater than +12 were shrunk to +12. Following the averaging operation and shrinking translation, each country was allocated one of the 25 'simplified' UTC zones ranging from UTC-12 through zulu time to UTC+12.

World population, countries, and development metrics

For peer review only - http://bmjopen.bmj.com/site/about/guidelines.xhtml

We used the United Nations World Population Prospects adjusted version of the 2020 population count data.[14] Each of the simplified global UTC offsets was then converted to a two-dimensional irregular polygon describing the shape of the area of the UTC offset, and the approximate population count in each zone was obtained by cross-referencing the global population count data within these polygons. We summed together the contributing areas of the global population count map within each simplified UTC offset polygon. We sought country count data from the United Nations Population Division.[15 16]

The human development index (HDI) is a national composite index using the geometric mean of scores for health assessed by life expectancy at birth, education measured by mean of years of schooling for adults aged ≥25 years and expected years of schooling for children of school entering age, and standard of living measured by gross national income per capita. We obtained national human development index (HDI) scores for the year 2019 from the United Nations Development Programme.[17] HDI scores were categorized as >0.800: very high; 0.700 - 0.799: high; 0.550 - 0.699: medium; and <0.550 low. Country and HDI data were cross-referenced with our country-to-simplified-UTC offset data set.

Health metrics

Disability adjusted life years (DALYs) are a societal measure of the burden of disease experienced by a population. DALYs are calculated as the sum of years of life lost from premature death, and years lost due to disability. Years lost due to disability are calculated by multiplying disease incidence by the disability weight, a measure of severity for the condition, by the duration of illness. Disability adjusted

For peer review only - http://bmjopen.bmj.com/site/about/guidelines.xhtml

life years (DALYs) per 100,000 for the year 2019, and for the illustrative diseases HIV/AIDS and typhoid and paratyphoid fever, were accessed through the Institute for Health Metrics and Evaluation Global Health Data Exchange.[18] DALY data were cross-referenced with our country-to-simplified-UTC offset data set.

Data visualisation

A world map was created in geographic coordinates using the World Geodetic System 1984 (WGS84) datum with colour-coded UTC offsets. UTC offset colour-coded bar plot panels were generated for each population and health metric corresponding to the map. In addition, an interactive three-dimensional UTC split-plot was created.

Temporal distance worked examples

To illustrate the impact of teleconference scheduling on participant temporal distance between two participants, we calculated temporal distance from a hypothetical call scheduled at noon in London, United Kingdom (UTC0); noon in Washington, DC, United States (UTC-5); and noon in New Delhi, India (UTC+5), to population and country count maxima, the median HDI minimum, and median DALY rate maxima UTC offsets identified through our spatial exploration.

Ethics framework

We considered the strengths and weaknesses of alternative strategies for scheduling international global health teleconferences in the context of ethics principles of distribution. These included egoism, which prioritises self-interest;[19] contractualism that holds an act to be wrong if its performance under the circumstances would be

For peer review only

disallowed by any set of principles for the general regulation of behaviour that no one could reasonably reject as a basis for informed, unforced, general agreement;[20] utilitarianism the view that the morally right action is the action that produces the most good;[21] egalitarianism, a school of thought that favours equality of some sort;[22] and prioritarianism, a view that the goodness of an outcome is a function not only of the overall well-being across all individuals, but with extra weight given to worse-off individuals.[23]

For peer review only

Patient and public involvement

In planning and designing this analysis, we gathered informally over two decades the perspectives of participants, and considered diverse strategies of organisers, of international teleconferences. Public involvement efforts intensified during COVID-19 pandemic travel restrictions.

RESULTS

World population, countries, and development by time zone

We demonstrated that the UTC offset world population maximum of approximately 1,724 million persons occurred in UTC+5, followed by UTC+8 at 1,630 million persons, and UTC+1 at 852 million persons. The world country count maximum of approximately 40 countries occurred in UTC+1, followed by UTC+2 at 26, and UTC+3 at 22. Of 203 countries classified, 20 (9.9%) were in time zones of Oceania, ranging from UTC+10 to UTC-7. The median human development index minimum of 0.527 occurred at UTC0, followed by UTC+11 at 0.588, and UTC+10 at 0.620 (Figure, Table 1).

For peer review only - http://bmjopen.bmj.com/site/about/guidelines.xhtml

World health by time zone

The UTC offset median total DALYs per 100,000 per year maximum of 41,873 occurred in UTC+11, followed by UTC0 at 37,563, and UTC+10 at 37,489. The UTC offset median HIV/AIDS DALYs per 100,000 per year maximum of 941 occurred at UTC0, followed by UTC-2 at 898, and UTC-5 at 696. By contrast, the UTC offset median typhoid and paratyphoid fevers DALYs per 100,000 per year maximum of 279 occurred at UTC+5, followed by UTC0 at 133, and UTC+7 at 119 (Figure, Table 1). An interactive three-dimensional plot of world population and health data by UTC offset is available at: https://www.stats.otago.ac.nz/~tdavies/utcsplit_health.html (allow up to 30 seconds to load in browser) and a static image is available as a Supplementary Figure.

Temporal distance worked examples

Table 2 shows temporal distances generated by hypothetical teleconferences scheduled for noon local time in London, United Kingdom; Washington, DC, United States; and New Delhi, India, to the identified UTC offset total population and total country count maximum, median HDI minimum, and median total and disease-specific DALY rate maxima. These simple, two-participant scenarios generated temporal distances of up to 11 hours to UTC offsets representing the countries count maximum and median DALYs per 100,000 per year maximum.

Ethics assessment

Table 3 summarises the strengths and weaknesses of alternative strategies for scheduling international teleconferences in global health in the context of ethics

For peer review only - http://bmjopen.bmj.com/site/about/guidelines.xhtml

principles of distribution. Egoism was identified as problematic and contractualism as unrealistically demanding for large teleconferences. Utilitarianism resulted in the potential for systematic disadvantage to small, temporally remote groups. Egalitarianism offered equality, but prioritarianism with its emphasis on offering explicit priority or precedence to disadvantaged or affected groups offered the possibility of addressing inequities.

DISCUSSION

Statement of principal findings

We demonstrate that the global population maximum, country count maximum, median HDI minimum, and the median total DALYs per 100,000 per year and median disease-specific DALYs per 100,000 per year maxima span a wide range of UTC offsets that can yield large temporal distances between international teleconference participants. In international teleconferences spanning most or all of the world's time zones, it is inevitable that some participants will be asked to join during the unfavourable window 11pm through 5am local time. But who should be asked to join during those hours and upon what basis? In order optimise representative participation and to support the health and wellbeing of attendees, we identify that teleconference scheduling based on egoism, and even utilitarian strategies that consistently favour the majority, is problematic. We suggest that at minimum egalitarian and ideally prioritarian approaches offering explicit priority or precedence to disadvantaged or affected groups be adopted in most circumstances.

Strengths and weaknesses of the study

For peer review only - http://bmjopen.bmj.com/site/about/guidelines.xhtml

To our knowledge, ours is the first attempt to display the world's population and health by time zone to inform teleconference scheduling in global health. While unique, our study had a number of limitations. We were unable to identify robust empiric data on the actual scheduling of global health conference calls and the impacts on participation and participants, so the premise for biases in teleconference scheduling was based on the opinion of the study team and public involvement garnered through fieldwork in the UTC+12 offset. We acknowledge that not all humans prefer to work during daylight hours, or to recreate. Because some time zones are offset from UTC by fractions of hours, at the time of writing there were 37 local times in use worldwide. For ease of presentation and interpretation we chose to 'shrink' time zones representing fractions of hours towards zero resulting in conflation of the 37 time zones to UTC and its 24 offsets. While this resulted in loss of 13 fractional time zones, we think that our approach reasonably approximated local apparent solar time, and thus corresponds well with the diurnal lifestyle of humans. Similarly, since countries with multiple time zones had contributing UTC values for population health metrics first averaged and then 'shrunk,' multiple time zone countries are represented in only single time zones. We considered that the resulting 'bunching' of metrics into single time zones was preferable to counting individual countries more than once. We recognise that population and health metrics by UTC offset are approximations based on limitations of source data and subsequent translations. Care should be taken when interpreting median values from UTC offsets that represent few countries, such as UTC+11 dominated by the Solomon Islands and Vanuatu. However, we do not anticipate that this had major impacts on rankings by UTC offset.

For peer review only - http://bmjopen.bmj.com/site/about/guidelines.xhtml

Comparison with other studies

We are not aware of similar research examining the world's population, countries, development, and health by UTC offset, and evaluating the implications of this from an ethics perspective. Nonetheless, our analysis contributes to broader discourse on the decolonisation of global health.[10 11] Coloniality in global health teleconferencing may be evident when calls on health conditions that most affect 'previously plundered nations' are led by, or scheduled to the preferences of, those in 'previously colonising nations' of the 'Global North.'[11 24] In the hypothetical worked examples, a prioritarian approach to scheduling a teleconference focused on HIV/AIDS would favour the global HIV/AIDS DALY rate maximum at UTC0. However, scheduling a teleconference on typhoid fever and paratyphoid fever based on egoism favouring the self-convenience of participants in Washington, DC, United States would create a large temporal distance of 10 hours to the global typhoid and paratyphoid fever DALY rate maximum at UTC+5. By contrast, temporal distance to those most affected by typhoid and paratyphoid fevers would be dramatically reduced by a prioritarian approach favouring daylight hours in New Delhi, India. Our spatial exploration by UTC offset could be extended to other diseases using publicly available datasets.

Implications for clinicians and policymakers

Persons asked to join international teleconference between 11pm and 5am may be less able or inclined to participate and thus be tacitly systematically excluded from global health discourse. Participants may experience circadian disruption known to have unfavourable impacts on wellbeing, longevity, and aging, as well as requiring night shift work, a probable human carginogen.[9] Decision making on who should be

For peer review only - http://bmjopen.bmj.com/site/about/guidelines.xhtml

inconvenienced may be considered as an application of the ethics of distribution. It would seem that there are few situations when egoism, when expressed as self-interest based on self-convenience, should be the primary driver of global health teleconference scheduling, as representative participation and global health goals compromised. Contractualism, while attractive as a means to incorporate a range of personal preferences, is highly demanding. For example, both self-regard and respect for others are required, and these may be in doubt when relationship ties are weak in large global health teleconferences that are not founded in established relationships.[3] Used without nuance, utilitarian methods using online scheduling tools designed to identify times convenient to the greatest number of potential participants may systematically disadvantage minority groups representing less populous UTC offsets, such as those of Oceania. This may be of particular concern if critical participants reside in these offsets, including those highly affected by the teleconference health condition of focus.

Egalitarian approaches to teleconference scheduling include systematically rotating UTC offsets to ensure that the burden of inconvenient times, or the ability to reasonably participate, is shared equally among attendees. An alternative egalitarian strategy is to adopt partial asynchrony, holding the same teleconference more than once at different UTC offsets. However, partial asynchrony compromises efficiency and precludes simultaneous, whole-group interaction. Whereas equality provides each individual or group of people the same resources and opportunities, equity recognises that each person has different circumstances, and allocates the resources and opportunities needed to reach an equal outcome.[25] Since global health strives for health equity,[1] we suggest that prioritarian approaches offering

For peer review only

explicit priority or precedence to disadvantaged or affected groups should be strongly considered in international teleconference scheduling. We acknowledge that this approach, favouring one group over others, may appear counterintuitive ethically. However, in this instance, it seeks to benefit those who are central to the health condition under investigation, and hence support their interests.

Conclusion

In conclusion, global health teleconferences may generate great temporal distances between participants that can compromise representative participation, as well as the wellbeing and health of attendees. In international teleconferencing spanning most or all of the world's time zones, some participants will inevitably be asked to participate between 11pm and 5am local time. While egalitarianism should be consider a minimum standard, our spatial exploration of the world's population and its health by UTC offset can be used to inform prioritarian approaches to scheduling international teleconferences offering explicit priority or precedence to disadvantaged or affected groups, in turn enhancing the global health aspiration to strive for health equity.

For peer review only

For peer review only - http://bmjopen.bmj.com/site/about/guidelines.xhtml

ACKNOWLEDGEMENTS

We thank Emeritus Professor D. Gareth Jones, MB BS, DSc, MD, Bioethics Centre, University of Otago, Dunedin, New Zealand, for critical review of the manuscript, focussing on the ethics framework and assessment.

We acknowledge the use of several public data sources for this project including the NASA Socioeconomic Data and Applications Center gridded population of the world version 4 for population density through Creative Commons Attribution international license 4.0 (https://creativecommons.org/licenses/by/4.0/); the United Nations Population Division for population and country data, and the United Nations Development Programme human development data center for human development index data, both through Creative Commons Attribution intergovernmental licence 3.0 (https://creativecommons.org/licenses/by/3.0/igo/legalcode); and the Institute for Health Metrics and Evaluation for health data through Creative Commons 4.0 attribution-non-commercial international licence 4.0 (https://creativecommons.org/licenses/by-nc/4.0/).

For peer review only

FOOTNOTES

Contributorship: JAC conceived the work. JAC and TMD designed the analysis. TMD undertook spatial exploration and created the figures. JAC did the temporal distance calculations and made the ethics assessment. JAC wrote the first draft of the manuscript, and TMD critically revised the manuscript for important intellectual content and gave final approval of the version to be published. JAC and TMD are the

For peer review only - http://bmjopen.bmj.com/site/about/guidelines.xhtml

guarantors. The corresponding author attests that both listed authors meet authorship criteria and that no others meeting the criteria have been omitted.

Funding: JAC received support from Bill & Melinda Gates Foundation grant OPP1151153.

Competing interests: All authors have completed the ICMJE uniform disclosure form at www.icmje.org/coi_disclosure.pdf and declare: no financial relationships with any organisations that might have an interest in the submitted work in the previous three years; no other relationships or activities that could appear to have influenced the submitted work.

Ethics approval: Not required.

Data sharing: Dataset, statistical code, and analytical methods are available via request to the corresponding author to other researchers for purposes of reproducing the results, replicating, or extending the analysis.

The lead author JAC affirms that the manuscript is an honest, accurate, and transparent account of the study being reported; that no important aspects of the study have been omitted; and that any discrepancies from the study as planned have been explained.

Dissemination to participants and related patient and public communities: Patients were not directly involved in the design and development of this study. As

For peer review only

this is a spatial exploration of publicly available data, no participant recruitment occurred. Dissemination will be via media release, social media, and through the University of Otago website.

Provenance and peer review: Not commissioned; externally peer reviewed.

For peer review only

For peer review only - http://bmjopen.bmj.com/site/about/guidelines.xhtml

Figure. Global population, countries, human development index, total and disease-specific disability adjusted life years per 100,000 by simplified Coordinated Universal Time offset, 2019-20

For peer review only

For peer review only - http://bmjopen.bmj.com/site/about/guidelines.xhtml

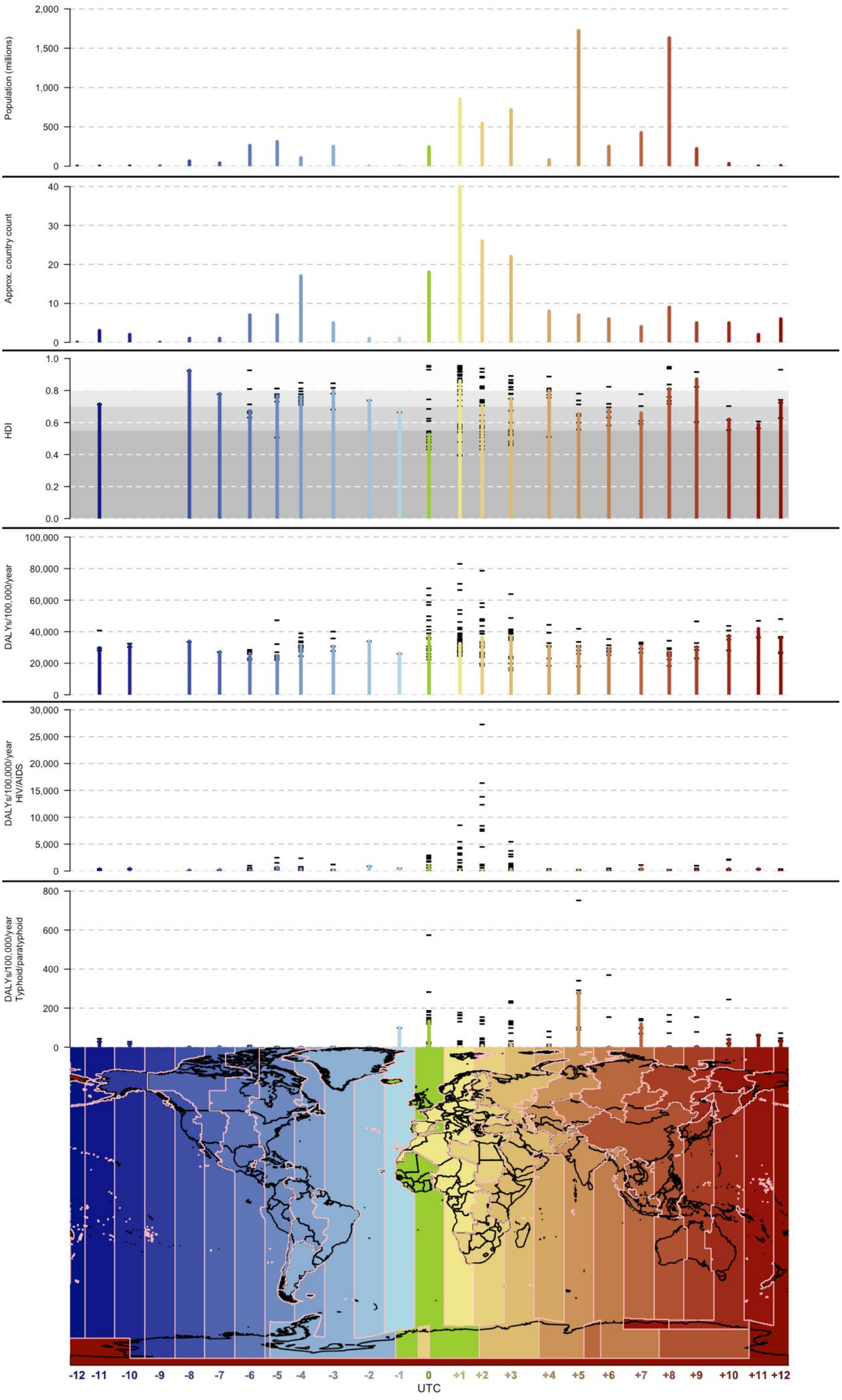
Population (millions)
Approx. country count
HDI
DALYs/100,000/year
DALYs/100,000/year HIV/AIDS
DALYs/100,000/year Typhoid/paratyphoid
-12 -11 -10 -9 -8 -7 -6 -5 -4 -3 -2 -1 0 +1 +2 +3 +4 +5 +6 +7 +8 +9 +10 +11 +12
UTC

HDI: Human development index; DALYs: disability-adjusted life years; UTC: Coordinated Universal Time.
Simplified Coordinated Universal Time offsets are colour-coded with pink lines at boundaries.
National borders are represented with black lines.
Plot bars show totals for population and country count.
Plot bars show median values for HDI and DALYs; black horizontal lines represent individual scores of each contributing country.
Grey-scale shading on the HDI plot represent categories of human development used by the United Nations Development Program: >0.800: very high; 0.700 - 0.799: high; 0.550 - 0.699: medium; and <0.550 low.

For peer review only

Table 1. Data summary of global population, countries, human development index, total and disease-specific disability adjusted life years per 100,000 by simplified Coordinated Universal Time offset, 2019-20

Characteristics		Coordinated Universal Time offset																							
		-12	-11	-10	-9	-8	-7	-6	-5	-4	-3	-2	-1	0	+1	+2	+3	+4	+5	+6	+7	+8	+9	+10	+11	+12
Population	Population, millions	0.00	0.08	1.80	0.63	64.35	40.43	261.90	311.67	106.33	252.00	0.14	1.60	244.65	851.90	545.55	716.67	81.13	1,723.74	251.18	424.72	1,630.13	221.12	32.81	2.14	6.49
	Country count	0	3	2	0	1	1	7	7	17	5	1	1	18	40	26	22	8	7	6	4	9	5	5	2	6
	HDI, median	.	0.715	.	.	0.926	0.779	0.673	0.767	0.762	0.800	0.738	0.665	0.527	0.860	0.709	0.750	0.800	0.654	0.683	0.659	0.810	0.871	0.620	0.588	0.734
Health	DALYs rate, total	.	29,768	31,485	.	33,866	27,197	26,108	24,875	30,834	30,884	33,899	26,307	37,563	32,428	36,295	36,965	30,970	30,538	29,078	30,841	26,871	30,353	37,489	41,872	36,348
	DALYs rate, HIV/AIDS	.	333.37	429.95	.	126.63	211.86	216.98	696.45	379.01	227.17	898.37	522.57	940.60	36.38	85.63	272.46	66.42	117.70	87.94	416.09	69.23	143.40	369.73	350.74	93.10
	DALYs rate, typhoid and paratyphoid	.	30.79	24.11	.	0.02	1.94	0.29	0.35	0.12	0.08	0.70	100.00	132.98	0.06	8.05	17.34	10.89	278.90	0.39	118.96	1.36	2.85	40.72	61.45	36.17

HDI: Human development index; DALYs rate: disability-adjusted life years per 100,000 per year; UTC: Coordinated Universal Time.

For peer review only

Table 2. Temporal distance from hypothetical teleconferences to key population and health Universal Coordinated Time offsets

			Temporal distance, hours*					
**Host city**	**Local time**	**UTC offset**	**Total population maximum**	**Country count maximum**	**Median HDI minimum**	**Median total DALY rate maximum**	**Median HIV/AIDS DALY rate maximum**	**Median typhoid and paratyphoid DALY rate maximum**
**Washington, DC, United States**	**Noon**	**-5**	**10**	**11**	**5**	**9**	**5**	**10**
**London, United Kingdom**	**Noon**	**0**	**5**	**1**	**0**	**11**	**0**	**5**
**New Delhi, India**	**Noon**	**+5**	**0**	**4**	**5**	**6**	**5**	**0**

*Shortest temporal distance in either easterly or westerly direction
UTC: Coordinated Universal Time; HDI: human development index; DALY rate: disability adjusted life years per 100,000 per year

For peer review only

Table 3. Ethics of distribution considerations in global health teleconference scheduling

Approach, reference	Summary	Scheduling priority	Strengths	Weaknesses
Egoism[19]	Maximise self-interest	Self-convenience	Limits disruption to the organiser	Compromises participation and wellbeing of invitees
Contractualism[20]	Wrong if disallowed by any set of principles for general regulation of behaviour	Self-regard and respect for others	Allows a variety of individual reasons to be considered	Highly demanding to implement, especially in large groups with weak ties
Utilitarianism[21]	Right action produces the most good	Convenient to the majority	Optimises the number of participants	Systematically disadvantages minority populations
Egalitarianism[22]	Favours equality of some sort	Inconvenience shared	Provides equal convenience and opportunity to participate	Insufficient to provide equity to disadvantaged groups
Prioritarianism[23]	Well-being across all individuals, with extra weight given to worse-off individuals	Priority to most affected, most hard-pressed, or least represented	Offers equity for disadvantaged groups	May yield fewer participants overall

For peer review only

Summary box

What is already known on this topic

- The COVID-19 pandemic has at once demanded increased levels of global health communication and coordination, and driven restrictions on face-to-face meetings resulting also in a marked increase in demand for international teleconferences.
- In international teleconferencing spanning most or all of the world's time zones, some will inevitably be asked to participate during the problematic time window of 11pm to 5am local time.
- Although there are considerable data on the world's population, countries, development, and health, these are conventionally reported by country precluding their consideration in international teleconference scheduling.

What this study adds

- We demonstrate that the global population maximum, country count maximum, median HDI minimum, and the median total DALYs per 100,000 per year and median disease-specific DALYs per 100,000 per year maxima span a wide range of UTC offsets that can yield large temporal distances between international teleconference participants
- We conclude that egalitarian approaches to teleconference scheduling are a minimum standard.
- Our spatial exploration of the world's population and its health by UTC offset can be used to inform prioritarian approaches to scheduling multiple time zone international teleconferences offering explicit priority or precedence to disadvantaged or affected groups, in turn enhancing the global health aspiration to strive for health equity

For peer review only - http://bmjopen.bmj.com/site/about/guidelines.xhtml

REFERENCES

1. Koplan JP, Bond TC, Merson MH, et al. Towards a common definition of global health. *Lancet* 2009;373:1993-5.

2. World Health Organisation. Coronavirus disease (COVID-19) advice for the public: World Health Organisation; 2021 [Available from: https://www.who.int/emergencies/diseases/novel-coronavirus-2019/advice-for-public accessed 17 June 2021.

3. Denstadli JM, Julsrud TE, Hjorthol RJ. Videoconferencing as a mode of communication: a comparative study of the use of videoconferencing and face-to-face meetings. *J Bus Tech Commun* 2012;26:65-91.

4. Amankwah-Amoah J. Stepping up and stepping out of COVID-19: new challenges for environmental sustainability policies in the global airline industry. *J Cleaner Production* 2020;271:123000.

5. International Conference held at Washington for the purpose of fixing a prime meridian and universal day October 1884. Protocols of the proceedings. Washington, DC, 1884.

6. Cummings JN. Geography is alive and well in virtual teams. *Commun ACM* 2011;54:24-6.

7. Aschoff J. Circadian rhythms in man. *Science* 1965;148:1427-32.

8. Morrison-Smith S, Ruiz J. Challenges and barriers in virtual teams: a literature review. *SN Appl Sci* 2020;2:1096.

9. IARC Monographs Vol 124 group. Carcinogenicity of night shift work. *Lancet Oncol* 2019;20:1058-9.

For peer review only - http://bmjopen.bmj.com/site/about/guidelines.xhtml

10. Abimbola S, Pai M. Will global health survive its decolonisation? *Lancet* 2020;396:1627-8.

11. Khan M, Abimbola S, Aloudat T, et al. Decolonising global health in 2021: a roadmap to move from rhetoric to reform. *BMJ Glob Health* 2021;6:e005604.

12. Teucher A. LUTZ: look up time zones of point coordinates. R package 0.3.1. 2019. https://CRAN.R-project.org/package=lutz (accessed 5 July 2021).

13. R Development Core Team. R: A language and environment for statistical computing. *R Foundation for Statistical Computing* 2019. https://www.R-project.org/ (accessed 5 July 2021).

14. Center for International Earth Science Information Network - CIESIN - Columbia University. Gridded population of the world, version 4 (GPWv4): population count adjusted to match 2015 revision of United Nations World Population Prospects country totals, revision 11. 2018. https://doi.org/10.7927/H4PN93PB (accessed 5 July 2021).

15. United Nations Department of Economic and Social Affairs Population Division. World population prospects 2019: volume I: comprehensive tables, 2019.

16. United Nations Department of Economic and Social Affairs Population Division. World population prospects 2019: volume II: demographic profiles, 2019.

17. United Nations Development Program. Human development reports: human development data center 2020 [Available from: http://hdr.undp.org/en/data accessed 5 July 2021.

18. Institute for Health Metrics and Evaluation. Global health data exchange Seattle, WA: IHME, University of Washington,; 2021 [Available from: http://ghdx.healthdata.org accessed 22 June 2021.

19. Sidgwick H. The methods of ethics. London: Macmillan 1874.

For peer review only - http://bmjopen.bmj.com/site/about/guidelines.xhtml

20. Scanlon TM. What we owe each other. Cambridge, MA: Hardvard University Press 1982.

21. Bentham J. An introduction to the principles of morals and legislation. Oxford: Clarendon Press, 1789.

22. Arneson R. Egalitarianism. In: Zalta EN, ed. The Stanford Encyclopedia of Philosophy. Stanford, CA, 2013.

23. Parfit D. Equality and priority. *Ratio* 2002;10:202-21.

24. Lees N. The Brandt Line after forty years: The more North–South relations change, the more they stay the same? *Rev Int Stud* 2021;47:85-106.

25. Braveman P, Gruskin S. Defining equity in health. *J Epidemiol Community Health* 2003;57:254-8.

For peer review only

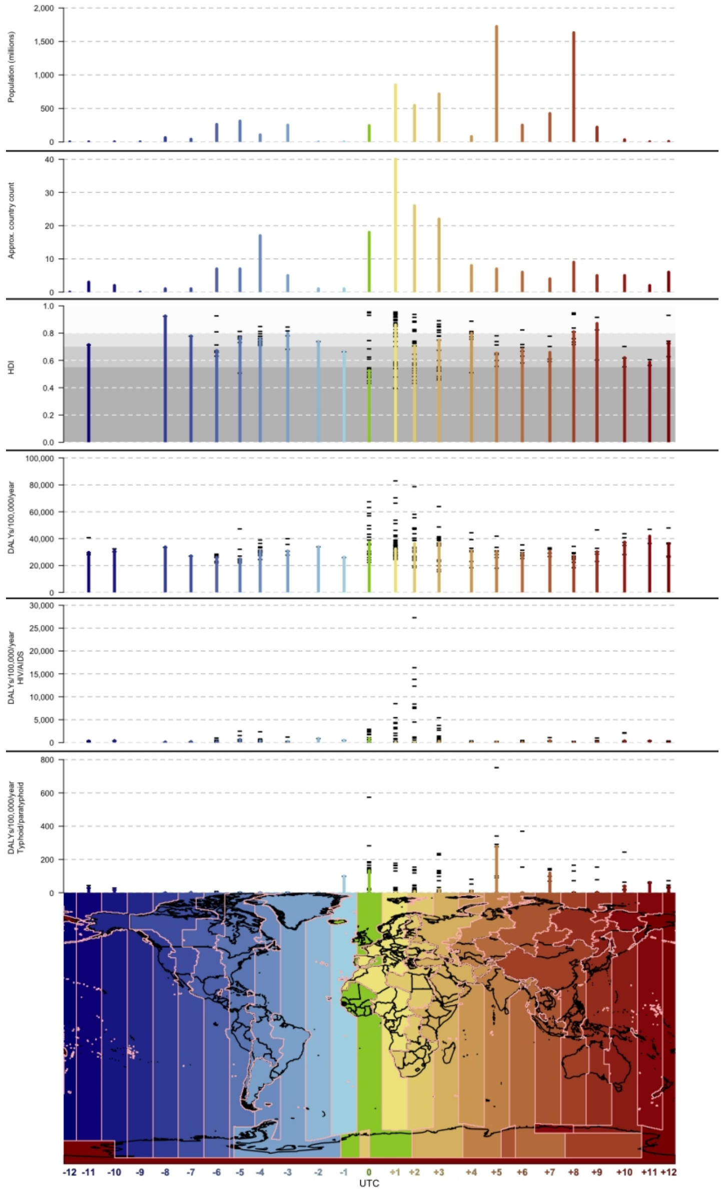

518x873mm (72 x 72 DPI)

Supplementary Figure. Static image of interactive three-dimensional UTC split-plot of global population, countries, human development index, total and disease-specific disability adjusted life years per 100,000 by simplified Coordinated Universal Time offset, 2019-20

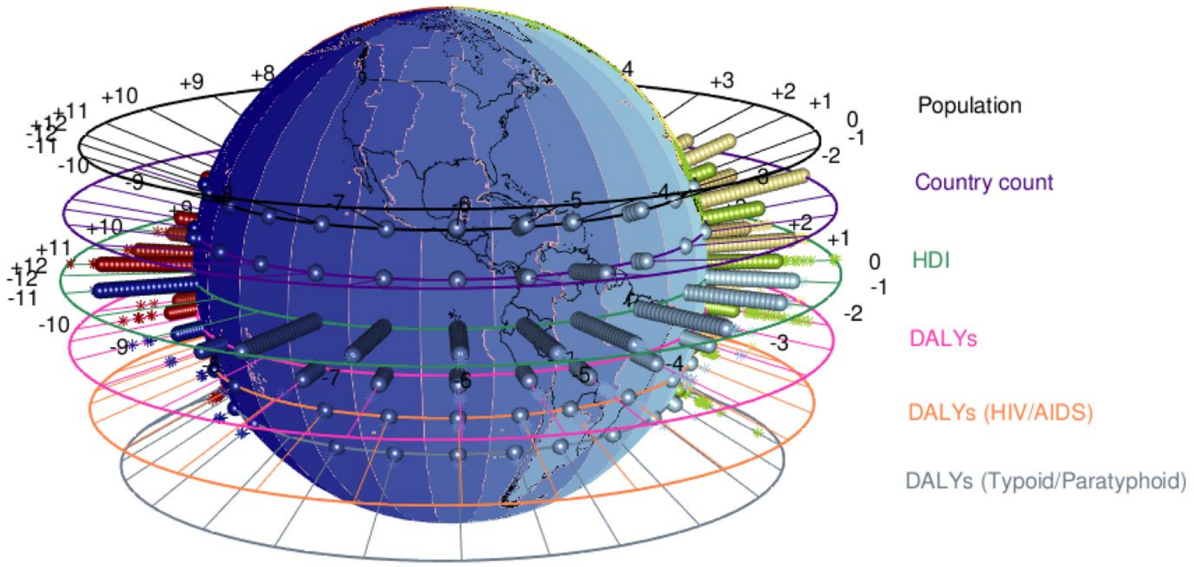

HDI: Human development index; DALYs: disability-adjusted life years; UTC: Coordinated Universal Time.
Interactive version available at:
https://www.stats.otago.ac.nz/~tdavies/utcsplit_health.html
(allow up to 30 seconds to load in browser)

For peer review only

STROBE Statement—checklist of items that should be included in reports of observational studies

	Item No	Recommendation	Page No
**Title and abstract**	1	(*a*) Indicate the study's design with a commonly used term in the title or the abstract	1
		(*b*) Provide in the abstract an informative and balanced summary of what was done and what was found	2-3
**Introduction**			
Background/rationale	2	Explain the scientific background and rationale for the investigation being reported	4-6
Objectives	3	State specific objectives, including any prespecified hypotheses	5-6
**Methods**			
Study design	4	Present key elements of study design early in the paper	6-9
Setting	5	Describe the setting, locations, and relevant dates, including periods of recruitment, exposure, follow-up, and data collection	6-9
Participants	6	(*a*) *Cohort study*—Give the eligibility criteria, and the sources and methods of selection of participants. Describe methods of follow-up<br>*Case-control study*—Give the eligibility criteria, and the sources and methods of case ascertainment and control selection. Give the rationale for the choice of cases and controls<br>*Cross-sectional study*—Give the eligibility criteria, and the sources and methods of selection of participants	6-9
		(*b*) *Cohort study*—For matched studies, give matching criteria and number of exposed and unexposed<br>*Case-control study*—For matched studies, give matching criteria and the number of controls per case	
Variables	7	Clearly define all outcomes, exposures, predictors, potential confounders, and effect modifiers. Give diagnostic criteria, if applicable	6-9
Data sources/ measurement	8*	For each variable of interest, give sources of data and details of methods of assessment (measurement). Describe comparability of assessment methods if there is more than one group	6-9
Bias	9	Describe any efforts to address potential sources of bias	6-9
Study size	10	Explain how the study size was arrived at	6-9
Quantitative variables	11	Explain how quantitative variables were handled in the analyses. If applicable, describe which groupings were chosen and why	6-9
Statistical methods	12	(*a*) Describe all statistical methods, including those used to control for confounding	6-9
		(*b*) Describe any methods used to examine subgroups and interactions	6-9
		(*c*) Explain how missing data were addressed	6-9
		(*d*) *Cohort study*—If applicable, explain how loss to follow-up was addressed<br>*Case-control study*—If applicable, explain how matching of cases and controls was addressed<br>*Cross-sectional study*—If applicable, describe analytical methods taking account of sampling strategy	6-9
		(*e*) Describe any sensitivity analyses	6-9

Continued on next page

For peer review only - http://bmjopen.bmj.com/site/about/guidelines.xhtml

**Results**			
Participants	13*	(a) Report numbers of individuals at each stage of study—eg numbers potentially eligible, examined for eligibility, confirmed eligible, included in the study, completing follow-up, and analysed	9-11
		(b) Give reasons for non-participation at each stage	NA
		(c) Consider use of a flow diagram	NA
Descriptive data	14*	(a) Give characteristics of study participants (eg demographic, clinical, social) and information on exposures and potential confounders	9-11
		(b) Indicate number of participants with missing data for each variable of interest	9-11
		(c) *Cohort study*—Summarise follow-up time (eg, average and total amount)	NA
Outcome data	15*	*Cohort study*—Report numbers of outcome events or summary measures over time	NA
		Case-control study—Report numbers in each exposure category, or summary measures of exposure	NA
		Cross-sectional study—Report numbers of outcome events or summary measures	9-11
Main results	16	(*a*) Give unadjusted estimates and, if applicable, confounder-adjusted estimates and their precision (eg, 95% confidence interval). Make clear which confounders were adjusted for and why they were included	NA
		(*b*) Report category boundaries when continuous variables were categorized	9-11
		(*c*) If relevant, consider translating estimates of relative risk into absolute risk for a meaningful time period	NA
Other analyses	17	Report other analyses done—eg analyses of subgroups and interactions, and sensitivity analyses	9-11
**Discussion**			
Key results	18	Summarise key results with reference to study objectives	11
Limitations	19	Discuss limitations of the study, taking into account sources of potential bias or imprecision. Discuss both direction and magnitude of any potential bias	11-12
Interpretation	20	Give a cautious overall interpretation of results considering objectives, limitations, multiplicity of analyses, results from similar studies, and other relevant evidence	12-15
Generalisability	21	Discuss the generalisability (external validity) of the study results	15
**Other information**			
Funding	22	Give the source of funding and the role of the funders for the present study and, if applicable, for the original study on which the present article is based	17

*Give information separately for cases and controls in case-control studies and, if applicable, for exposed and unexposed groups in cohort and cross-sectional studies.

Note: An Explanation and Elaboration article discusses each checklist item and gives methodological background and published examples of transparent reporting. The STROBE checklist is best used in conjunction with this article (freely available on the Web sites of PLoS Medicine at http://www.plosmedicine.org/, Annals of Internal Medicine at http://www.annals.org/, and Epidemiology at http://www.epidem.com/). Information on the STROBE Initiative is available at www.strobe-statement.org.

For peer review only

For peer review only - http://bmjopen.bmj.com/site/about/guidelines.xhtml

BMJ Open

Towards equitable scheduling of global health teleconferences: a spatial exploration of the world's population and health by time zone

Journal:	*BMJ Open*
Manuscript ID	bmjopen-2021-056696.R1
Article Type:	Original research
Date Submitted by the Author:	19-Feb-2022
Complete List of Authors:	Crump, John; University of Otago, Centre for International Health<br>Davies, Tilman ; University of Otago, Department of Mathematics and Statistics
**Primary Subject Heading**:	Global health
Secondary Subject Heading:	Communication, Epidemiology, Ethics, Health policy, Occupational and environmental medicine
Keywords:	MEDICAL ETHICS, Telemedicine < BIOTECHNOLOGY & BIOINFORMATICS, EPIDEMIOLOGY, International health services < HEALTH SERVICES ADMINISTRATION & MANAGEMENT, OCCUPATIONAL & INDUSTRIAL MEDICINE, STATISTICS & RESEARCH METHODS

SCHOLARONE™
Manuscripts

I, the Submitting Author has the right to grant and does grant on behalf of all authors of the Work (as defined in the below author licence), an exclusive licence and/or a non-exclusive licence for contributions from authors who are: i) UK Crown employees; ii) where BMJ has agreed a CC-BY licence shall apply, and/or iii) in accordance with the terms applicable for US Federal Government officers or employees acting as part of their official duties; on a worldwide, perpetual, irrevocable, royalty-free basis to BMJ Publishing Group Ltd ("BMJ") its licensees and where the relevant Journal is co-owned by BMJ to the co-owners of the Journal, to publish the Work in this journal and any other BMJ products and to exploit all rights, as set out in our licence.

The Submitting Author accepts and understands that any supply made under these terms is made by BMJ to the Submitting Author unless you are acting as an employee on behalf of your employer or a postgraduate student of an affiliated institution which is paying any applicable article publishing charge ("APC") for Open Access articles. Where the Submitting Author wishes to make the Work available on an Open Access basis (and intends to pay the relevant APC), the terms of reuse of such Open Access shall be governed by a Creative Commons licence – details of these licences and which Creative Commons licence will apply to this Work are set out in our licence referred to above.

Other than as permitted in any relevant BMJ Author's Self Archiving Policies, I confirm this Work has not been accepted for publication elsewhere, is not being considered for publication elsewhere and does not duplicate material already published. I confirm all authors consent to publication of this Work and authorise the granting of this licence.

For peer review only

Towards equitable scheduling of global health teleconferences: a spatial exploration of the world's population and health by time zone

John A. Crump, MB ChB, MD, DTM&H[1]

Tilman M. Davies, PhD[2]

[1]Centre for International Health, University of Otago, Dunedin, New Zealand

[2]Department of Mathematics and Statistics, University of Otago, Dunedin, New Zealand

Corresponding author: Prof. John A. Crump, Centre for International Health, University of Otago, PO Box 56, Dunedin, 9054, New Zealand. Tel +64-3-479-9460, Email: john.crump@otago.ac.nz

For peer review only

Word count: 3,199

ABSTRACT

Objectives To examine the world's population, development, and health by condensed Coordinated Universal Time (UTC) offset to support a normative position on how to most defensibly schedule global health teleconferences with the primary goal of advancing equitable regard for participants by health condition.

Design Spatial exploration examined through the lens of equitable regard for participants.

Setting The earth.

Participants The global population.

Main outcome measures Global population, countries, human development index, and health measured in total and disease-specific disability adjusted life years (DALYs) per 100,000 per year by UTC offset. Strengths and weaknesses of scheduling teleconferences accordingly to alternative approaches.

Results The UTC offset world population maximum of approximately 1,724 million persons occurred in UTC+5, country count maximum of approximately 40 countries in UTC+1, and the median human development index minimum of 0.527 occurred at UTC0. The median total DALYs per 100,000 per year maximum of 41,873 occurred in UTC+11, median HIV/AIDS DALYs per 100,000 per year maximum of 941 in UTC0, and median typhoid and paratyphoid fevers DALYs per 100,000 per year

For peer review only

maximum of 279 occurred at UTC+5. Hypothetical teleconference scheduling scenarios generated temporal distances of up to 11 hours to UTC offsets representing the most countries and greatest number of DALYs per 100,000 per year. Teleconference scheduling based on egoism was considered to be problematic, and contractualism as unrealistically demanding. Utilitarianism resulted in the risk of systematically disadvantaging small, temporally remote groups. Egalitarianism offered equality, but prioritarianism offered the possibility of addressing health inequities.

Conclusions Global health teleconferences may generate great temporal distances between participants compromising representative participation, as well as the wellbeing of attendees. Our spatial exploration of the world's population and health by UTC offset may be used to support a normative position on scheduling global health teleconferences that enhance progress towards health equity.

For peer review only

For peer review only - http://bmjopen.bmj.com/site/about/guidelines.xhtml

ARTICLE SUMMARY

Strengths and limitations of this study

- We displayed the world's population and health by time zone to inform a normative position on scheduling global health teleconferences that span many or most global time zones.
- Inequities in teleconference scheduled were illustrated with hypothetical scenarios through calculating temporal distance between two participants in relation to population and country count maxima, the median human development index minimum, and median total and disease-specific disability adjusted life year rate maxima Coordinated Universal Time offsets.
- An ethics assessment of the strengths and weaknesses of alternative strategies for scheduling international teleconferences in global health was made in the context of ethics principles of distribution, examining stragies based in egoism, contractualism, utilitarianism, egalitarianism, and prioritarianism.

For peer review only

INTRODUCTION

Addressing global health problems through collaboration depends in part on international synchronous communication and coordination.[1] In modern times, this is typically achieved by teleconference, a collective term for communication using a range of platforms including voice only conference calls, videoconferences, and web-based meetings. Addressing the COVID-19 pandemic has at once demanded increased levels of global health communication and coordination, and driven restrictions on face-to-face meetings[2] resulting also in a marked increase in demand for international teleconferences for communication on a wide range of health conditions. While teleconferences have been considered inferior to face-to-face meetings for complex tasks with high ambiguity and where new ties need to be built,[3] the aspiration to pursue more judicious use of cleaner international air travel following the COVID-19 pandemic[4] point to a future with a sustained high level of international teleconference use.

Since the earth approximate a sphere that orbits the sun and spins, daylight is experienced in sequence by longitude on a 24 hour cycle. To manage this, the world is presently organised into 37 longitudinal time zones adapted to follow national and subnational boundaries, and observed as Coordinated Universal Time (UTC) offsets from the prime meridian in Greenwich, England, the international standard for zero degrees longitude,[5] and home to UTC offset zero or 'zulu time.' The value of being home to the prime meridian was reflected in the 'scramble' among predominantly western nations to become the host, a matter that was resolved at a conference in Washington, DC, in the United States in 1884.[5] Among the many consequences of this decision experienced today, participants joining teleconferences at locations

For peer review only

remote to zulu time may be more likely to be asked to participate between the hours of 11pm through 5am local time, a time window classified as unfavourable by some online teleconference scheduling tools, and a manifestation of a form of discrimination termed antimeridianism.[6]

Temporal distance is a measure, distinct from geographic distance, defined as a directional measurement of the temporal displacement experienced by two collaborators who want to interact with each other.[7] Because humans are a diurnal species with a circadian clock entrained closely to light, traditionally from the sun,[8] the temporal distance inherent in synchronous multiple time zone teleconferencing poses a major challenge to representative participation[9] and to supporting the wellbeing of attendees.[10] Methods in common use for scheduling international conference calls include online tools facilitating identification of times convenient to the greatest number of potential participants, a form of utilitarianism. However, scheduling may be influenced by other drivers including egoism, where the preferences of the organiser are prioritised. In teleconference spanning many or most global time zones, some participants will be inevitably be asked to join between the hours of 11pm through 5am local time. But who should be asked to join at these times and why? While global health claims to seek health equity,[1] it is recognized that aspects of its history in colonial medicine, tropical medicine, and international health may perpetuate inequities.[11] The decolonising global health movement 'fights against ingrained systems of dominance and power in the work to improve the health of populations, whether this occurs between countries, including between previously colonising and plundered nations, and within countries.'[12] To our knowledge, there

For peer review only

has hitherto been little specific attention given to supporting equity in the organisation and scheduling of international teleconferences in global health.

In order to inform equitable approaches to scheduling multiple time zone teleconferences in global health, we sought to describe empirical data on the world's population and its health by time zone. The primary objectives of this analysis were to display human population characteristics and health by time zone and to consider the implications of these findings in the context of the ethics of distribution, an area of ethics that addresses the distribution of benefits and burdens across separate persons or groups. We aspired to identify a normative position to enhance equitable organisation and participation, and ultimately outcomes, for teleconferences in global health.

METHODS

We explored the world's population and its health by time zone. We illustrated the implications of the findings with worked examples, and assessed alternative strategies for international teleconference scheduling in light of the ethics of distribution with the primary goal of advancing equitable regard for participants by health condition.

Time zones

We constructed a fine grid of longitude coordinates ranging from -180º to 180º and of latitude coordinates ranging from -90º to 90º and fed the grid into the lutz ('look up time zones') software[13] in the R programming environment.[14] Each coordinate was

For peer review only - http://bmjopen.bmj.com/site/about/guidelines.xhtml

linked to a country and region identifier, and to a non-daylight savings time UTC offset value. For simplicity, fractional UTC offsets were 'shrunk' towards zero. For example, UTC+8.5 translated to UTC+8, and UTC-3.5 to UTC-3. For countries with multiple time zones, their contributing UTC values were first averaged, and then the shrinking translation was performed. Multiple time zone country data were assigned to that single shrunk time zone. A small number of countries with UTC offsets greater than +12 were shrunk to +12. Following the averaging operation and shrinking translation, each country was allocated one of the 25 'simplified' UTC zones ranging from UTC-12 through zulu time to UTC+12.

World population, countries, and development metrics

We used the United Nations World Population Prospects adjusted version of the 2020 population count data.[15] Each of the simplified global UTC offsets was then converted to a two-dimensional irregular polygon describing the shape of the area of the UTC offset, and the approximate population count in each zone was obtained by cross-referencing the global population count data within these polygons. We summed together the contributing areas of the global population count map within each simplified UTC offset polygon. We sought country count data from the United Nations Population Division.[16 17]

The human development index (HDI) is a national composite index using the geometric mean of scores for health assessed by life expectancy at birth, education measured by mean of years of schooling for adults aged ≥25 years and expected years of schooling for children of school entering age, and standard of living measured by gross national income *per capita*. We obtained national human

For peer review only

development index (HDI) scores for the year 2019 from the United Nations Development Programme.[18] HDI scores were categorized as >0.800: very high; 0.700 - 0.799: high; 0.550 - 0.699: medium; and <0.550 low. Country and HDI data were cross-referenced with our country-to-simplified-UTC offset data set.

Health metrics

Disability adjusted life years (DALYs) are a societal measure of the burden of disease experienced by a population. DALYs are calculated as the sum of years of life lost from premature death, and years lost due to disability. Years lost due to disability are calculated by multiplying disease incidence by the disability weight, a measure of severity for the condition, by the duration of illness. Disability adjusted life years (DALYs) per 100,000 for the year 2019, and for the illustrative diseases HIV/AIDS and typhoid and paratyphoid fever, were accessed through the Institute for Health Metrics and Evaluation Global Health Data Exchange.[19] DALY data were cross-referenced with our country-to-simplified-UTC offset data set.

Data visualisation

A world map was created in geographic coordinates using the World Geodetic System 1984 (WGS84) datum with colour-coded UTC offsets. UTC offset colour-coded bar plot panels were generated for each population and health metric corresponding to the map. In addition, an interactive three-dimensional UTC split-plot was created.

Temporal distance worked examples

For peer review only

To illustrate the impact of teleconference scheduling on participant temporal distance between two participants, we calculated temporal distance from a hypothetical call scheduled at noon in London, United Kingdom (UTC0); noon in Washington, DC, United States (UTC-5); and noon in New Delhi, India (UTC+5), to population and country count maxima, the median HDI minimum, and median DALY rate maxima UTC offsets identified through our spatial exploration.

Ethics framework

To support a normative position, we considered the strengths and weaknesses of alternative strategies for scheduling international global health teleconferences in the context of ethics principles of distribution, an area of ethics that addresses how the benefits and burdens are distributed across separate persons or groups. These included egoism, which prioritises self-interest;[20] contractualism that holds an act to be wrong if its performance under the circumstances would be disallowed by any set of principles for the general regulation of behaviour that no one could reasonably reject as a basis for informed, unforced, general agreement;[21] utilitarianism the view that the morally right action is the action that produces the most good;[22] egalitarianism, a school of thought that favours equality of some sort;[23] and prioritarianism, a view that the goodness of an outcome is a function not only of the overall well-being across all individuals, but with extra weight given to worse-off individuals.[24]

Patient and public involvement

Our research was informed by two decades of global health teleconference participation, including from a low- and middle-income country perspective in

For peer review only

Tanzania and from the perspectives of participation from New Zealand in UTC+12. Interest in the topic was intensified during COVID-19 pandemic travel restrictions.

RESULTS

World population, countries, and development by time zone

We demonstrated that the UTC offset world population maximum of approximately 1,724 million persons occurred in UTC+5, followed by UTC+8 at 1,630 million persons, and UTC+1 at 852 million persons. The world country count maximum of approximately 40 countries occurred in UTC+1, followed by UTC+2 at 26, and UTC+3 at 22. Of 203 countries classified, 20 (9.9%) were in time zones of Oceania, ranging from UTC+10 to UTC-7. The median human development index minimum of 0.527 occurred at UTC0, followed by UTC+11 at 0.588, and UTC+10 at 0.620 (Figure, Table 1).

World health by time zone

The UTC offset median total DALYs per 100,000 per year maximum of 41,873 occurred in UTC+11, followed by UTC0 at 37,563, and UTC+10 at 37,489. The UTC offset median HIV/AIDS DALYs per 100,000 per year maximum of 941 occurred at UTC0, followed by UTC-2 at 898, and UTC-5 at 696. By contrast, the UTC offset median typhoid and paratyphoid fevers DALYs per 100,000 per year maximum of 279 occurred at UTC+5, followed by UTC0 at 133, and UTC+7 at 119 (Figure, Table 1). An interactive three-dimensional plot of world population and health data by UTC offset is available at: https://www.stats.otago.ac.nz/~tdavies/utcsplit_health.html

For peer review only

(allow up to 30 seconds to load in browser) and a static image is available as a Supplementary Figure.

Temporal distance worked examples

Table 2 shows temporal distances generated by hypothetical teleconferences scheduled for noon local time in London, United Kingdom; Washington, DC, United States; and New Delhi, India, to the identified UTC offset total population and total country count maximum, median HDI minimum, and median total and disease-specific DALY rate maxima. These simple, two-participant scenarios generated temporal distances of up to 11 hours to UTC offsets representing the countries count maximum and median DALYs per 100,000 per year maximum.

Ethics assessment

Table 3 summarises the strengths and weaknesses of alternative strategies for scheduling international teleconferences in global health in the context of ethics principles of distribution. Egoism was identified as problematic and contractualism as unrealistically demanding for large teleconferences. Utilitarianism resulted in the potential for systematic disadvantage to small, temporally remote groups. Egalitarianism offered equality, but prioritarianism with its emphasis on offering explicit priority or precedence to disadvantaged or affected groups offered the possibility of addressing inequities.

DISCUSSION

Statement of principal findings

For peer review only

We demonstrate that the global population maximum, country count maximum, median HDI minimum, and the median total DALYs per 100,000 per year and median disease-specific DALYs per 100,000 per year maxima span a wide range of UTC offsets that can yield large temporal distances between international teleconference participants. In international teleconferences spanning many or most of the world's time zones, it is inevitable that some participants will be asked to join during the unfavourable window 11pm through 5am local time. But who should be asked to join during those hours and upon what basis? In order optimise representative participation and to support the wellbeing of attendees, we identify that teleconference scheduling based on egoism, and even utilitarian strategies that consistently favour the majority, is problematic. We suggest that at minimum egalitarian, and ideally prioritarian, approaches offering explicit priority or precedence to disadvantaged or affected groups be adopted in most circumstances.

Strengths and weaknesses of the study

To our knowledge, ours is the first attempt to display the world's population and health by time zone to inform teleconference scheduling in global health. While unique, our study had a number of limitations. We were unable to identify robust empiric data on the actual scheduling of global health conference calls and the impacts on participation and participants, so the premise for biases in teleconference scheduling was based on the qualitative experience of the study team and public involvement garnered through fieldwork in a low- and middle-income country and in the UTC+12 offset. We acknowledge that not all humans prefer to work during daylight hours, or to recreate. Because some time zones are offset from UTC by fractions of hours, at the time of writing there were 37 local times in use worldwide.

For peer review only - http://bmjopen.bmj.com/site/about/guidelines.xhtml

For ease of presentation and interpretation we chose to ‘shrink’ time zones representing fractions of hours towards zero resulting in conflation of the 37 time zones to UTC and its 24 offsets. While this resulted in loss of 13 fractional time zones, we think that our approach reasonably approximated local apparent solar time, and thus corresponds well with the diurnal lifestyle of humans. Similarly, since countries with multiple time zones had contributing UTC values for population health metrics first averaged and then ‘shrunk,’ multiple time zone countries are represented in only single time zones. We considered that the resulting ‘bunching’ of metrics into single time zones was preferable to counting individual countries more than once. We recognise that UTC offset is an imperfect surrogate for local apparent solar time, a superior but less practical benchmark for measuring inconvenience in relation to the human circadian cycle. Further, population and health metrics by UTC offset are approximations based on limitations of source data and subsequent translations. Care should be taken when interpreting median values from UTC offsets that represent few countries, such as UTC+11 dominated by the Solomon Islands and Vanuatu. However, we do not anticipate that this had major impacts on rankings by UTC offset. We also acknowledge that factors beyond those contributing to equity of participation may be relevant in teleconference scheduling, but explicit consideration of these was beyond the scope of our analysis.

Comparison with other studies

We are not aware of similar research examining the world’s population, countries, development, and health by UTC offset, nor of work attempting to support a normative position on how to most defensibly schedule global health teleconferences with the primary goal of advancing equitable regard for participants by health

For peer review only

condition. Nonetheless, our analysis contributes to broader discourse on the decolonisation of global health.[11 12] Coloniality in global health teleconferencing may be evident when calls on health conditions that most affect ‘previously plundered nations’ are led by, or scheduled to the preferences of, those in ‘previously colonising nations’ of the ‘Global North.’[12 25] In the hypothetical worked examples, a prioritarian approach to scheduling a teleconference focused on HIV/AIDS would favour the global HIV/AIDS DALY rate maximum at UTC0. However, scheduling a teleconference on typhoid fever and paratyphoid fever based on egoism favouring the self-convenience of participants in Washington, DC, United States would create a large temporal distance of 10 hours to the global typhoid and paratyphoid fever DALY rate maximum at UTC+5. By contrast, temporal distance to those most affected by typhoid and paratyphoid fevers would be dramatically reduced by a prioritarian approach favouring daylight hours in New Delhi, India. Our spatial exploration by UTC offset could be extended to other diseases using publicly available datasets.

Implications for clinicians and policymakers

Persons asked to join international teleconference between 11pm and 5am may be less able or inclined to participate and thus be tacitly systematically excluded from global health discourse, a manifestation of a form of discrimination termed antimeridianism. Such participants may experience circadian disruption known to have unfavourable impacts on wellbeing.[10] Decision making on who should be inconvenienced may be considered an application of the ethics of distribution. It would seem that there are few situations when egoism, when expressed as self-interest based on self-convenience, should be the primary driver of global health

For peer review only - http://bmjopen.bmj.com/site/about/guidelines.xhtml

teleconference scheduling, as representative participation and global health goals compromised. Contractualism, while attractive as a means to incorporate a range of personal preferences, is highly demanding. For example, both self-regard and respect for others are required, and these may be in doubt when social ties are weak in large global health teleconferences that are not founded in established relationships.[3] Used without nuance, utilitarian methods using online scheduling tools designed to identify times convenient to the greatest number of potential participants may systematically disadvantage minority groups representing less populous UTC offsets, such as those of Oceania. This may be of particular concern if critical participants reside in these offsets, including those highly affected by the teleconference health condition of focus.

Egalitarian approaches to teleconference scheduling include systematically rotating UTC offsets to ensure that the burden of inconvenient times, or the ability to reasonably participate, is shared equally among attendees. An alternative egalitarian strategy is to adopt partial asynchrony, holding the same teleconference more than once at different UTC offsets. However, partial asynchrony compromises efficiency and precludes simultaneous, whole-group interaction. Whereas equality provides each individual or group of people the same resources and opportunities, equity recognises that each person has different circumstances, and allocates the resources and opportunities needed to reach an equal outcome.[26] Since global health strives for health equity,[1] we suggest that prioritarian approaches offering explicit priority or precedence to disadvantaged or affected groups should be strongly considered in international teleconference scheduling. We acknowledge that this approach, favouring one group over others, may appear counterintuitive

For peer review only - http://bmjopen.bmj.com/site/about/guidelines.xhtml

ethically. However, in this instance, it seeks to benefit those who are central to the health condition under investigation, and hence support their interests.

Finally, there are many domains of global health in which moral rhetoric is not matched by practice.[27] We hope that our pragmatic analysis and normative position might be an exception and that our findings can be translated in better practice in teleconference scheduling by clinicians and policymakers in the global health field.

Conclusion

In conclusion, while just one of a range of fundamental moral and ethical inconsistencies in global health, teleconferences may generate great temporal distances between participants that can compromise representative participation, as well as the wellbeing of attendees. In international teleconferencing spanning many or most of the world's time zones, some participants will inevitably be asked to participate between 11pm and 5am local time. While egalitarianism should be consider a minimum standard, our spatial exploration of the world's population and its health by UTC offset can be used to inform prioritarian approaches to scheduling international teleconferences offering explicit priority or precedence to disadvantaged or affected groups, in turn enhancing the global health aspiration to strive for health equity. We suggest that practical tools be designed to display global population, countries, human development index, and health metrics by condition by UTC offset to inform global health teleconference scheduling.

For peer review only

ACKNOWLEDGEMENTS

We thank Emeritus Professor D. Gareth Jones, MB BS, DSc, MD, Bioethics Centre, University of Otago, Dunedin, New Zealand, for critical review of the manuscript, focussing on the ethics framework and assessment.

We acknowledge the use of several public data sources for this project including the NASA Socioeconomic Data and Applications Center gridded population of the world version 4 for population density through Creative Commons Attribution international license 4.0 (https://creativecommons.org/licenses/by/4.0/); the United Nations Population Division for population and country data, and the United Nations Development Programme human development data center for human development index data, both through Creative Commons Attribution intergovernmental licence 3.0 (https://creativecommons.org/licenses/by/3.0/igo/legalcode); and the Institute for Health Metrics and Evaluation for health data through Creative Commons 4.0 attribution-non-commercial international licence 4.0 (https://creativecommons.org/licenses/by-nc/4.0/).

FOOTNOTES

Contributorship: JAC conceived the work. JAC and TMD designed the analysis. TMD undertook spatial exploration and created the figures. JAC did the temporal distance calculations and made the ethics assessment. JAC wrote the first draft of the manuscript, and TMD critically revised the manuscript for important intellectual content and gave final approval of the version to be published. JAC and TMD are the

For peer review only

guarantors. The corresponding author attests that both listed authors meet authorship criteria and that no others meeting the criteria have been omitted.

Funding: JAC received support from Bill & Melinda Gates Foundation grant OPP1151153.

Competing interests: All authors have completed the ICMJE uniform disclosure form at www.icmje.org/coi_disclosure.pdf and declare: no financial relationships with any organisations that might have an interest in the submitted work in the previous three years; no other relationships or activities that could appear to have influenced the submitted work.

Ethics approval: Not required.

Data sharing: Dataset, statistical code, and analytical methods are available via request to the corresponding author to other researchers for purposes of reproducing the results, replicating, or extending the analysis.

The lead author JAC affirms that the manuscript is an honest, accurate, and transparent account of the study being reported; that no important aspects of the study have been omitted; and that any discrepancies from the study as planned have been explained.

Dissemination to participants and related patient and public communities: Patients were not directly involved in the design and development of this study. As

For peer review only

this is a spatial exploration of publicly available data, no participant recruitment occurred. Dissemination will be via media release, social media, and through the University of Otago website.

Provenance and peer review: Not commissioned; externally peer reviewed.

For peer review only

Figure. Global population, countries, human development index, total and disease-specific disability adjusted life years per 100,000 by simplified Coordinated Universal Time offset, 2019-20

HDI: Human development index; DALYs: disability-adjusted life years; UTC: Coordinated Universal Time.
Simplified Coordinated Universal Time offsets are colour-coded with pink lines at boundaries.
National borders are represented with black lines.
Plot bars show totals for population and country count.
Plot bars show median values for HDI and DALYs; black horizontal lines represent individual scores of each contributing country.
Grey-scale shading on the HDI plot represent categories of human development used by the United Nations Development Program: >0.800: very high; 0.700 - 0.799: high; 0.550 - 0.699: medium; and <0.550 low.

For peer review only

Table 1. Data summary of global population, countries, human development index, total and disease-specific disability adjusted life years per 100,000 by simplified Coordinated Universal Time offset, 2019-20

Characteristics		Coordinated Universal Time offset																								
		-12	-11	-10	-9	-8	-7	-6	-5	-4	-3	-2	-1	0	+1	+2	+3	+4	+5	+6	+7	+8	+9	+10	+11	+12
Population	Population, millions	0.00	0.08	1.80	0.63	64.35	40.43	261.90	311.67	106.33	252.00	0.14	1.60	244.65	851.90	545.55	716.67	81.13	1,723.74	251.18	424.72	1,630.13	221.12	32.81	2.14	6.49
	Country count	0	3	2	0	1	1	7	7	17	5	1	1	18	40	26	22	8	7	6	4	9	5	5	2	6
	HDI, median	.	0.715	.	.	0.926	0.779	0.673	0.767	0.762	0.800	0.738	0.665	0.527	0.860	0.709	0.750	0.800	0.654	0.683	0.659	0.810	0.871	0.620	0.588	0.734
Health	DALYs rate, total	.	29,768	31,485	.	33,866	27,197	26,108	24,875	30,834	30,884	33,899	26,307	37,563	32,428	36,295	36,965	30,970	30,538	29,078	30,841	26,871	30,353	37,489	41,872	36,348
	DALYs rate, HIV/AIDS	.	333.37	429.95	.	126.63	211.86	216.98	696.45	379.01	227.17	898.37	522.57	940.60	36.38	85.63	272.46	66.42	117.70	87.94	416.09	69.23	143.40	369.73	350.74	93.10
	DALYs rate, typhoid and paratyphoid	.	30.79	24.11	.	0.02	1.94	0.29	0.35	0.12	0.08	0.70	100.00	132.98	0.06	8.05	17.34	10.89	278.90	0.39	118.96	1.36	2.85	40.72	61.45	36.17

HDI: Human development index; DALYs rate: disability-adjusted life years per 100,000 per year; UTC: Coordinated Universal Time.

For peer review only

2

Table 2. Temporal distance generated in hypothetical teleconferences to key population and health Universal Coordinated Time offsets

			Temporal distance, hours*					
**Host city**	**Local time**	**UTC offset**	**Total population maximum**	**Country count maximum**	**Median HDI minimum**	**Median total DALY rate maximum**	**Median HIV/AIDS DALY rate maximum**	**Median typhoid and paratyphoid DALY rate maximum**
**Washington, DC, United States**	**Noon**	**-5**	**10**	**11**	**5**	**9**	**5**	**10**
**London, United Kingdom**	**Noon**	**0**	**5**	**1**	**0**	**11**	**0**	**5**
**New Delhi, India**	**Noon**	**+5**	**0**	**4**	**5**	**6**	**5**	**0**

*Shortest temporal distance in either easterly or westerly direction
UTC: Coordinated Universal Time; HDI: human development index; DALY rate: disability adjusted life years per 100,000 per year

For peer review only

For peer review only - http://bmjopen.bmj.com/site/about/guidelines.xhtml

Table 3. Ethics of distribution considerations in global health teleconference scheduling

Approach, reference	Summary	Scheduling priority	Strengths	Weaknesses
Egoism[20]	Maximise self-interest	Self-convenience	Limits disruption to the organiser	Compromises participation and wellbeing of invitees
Contractualism[21]	Wrong if disallowed by any set of principles for general regulation of behaviour	Self-regard and respect for others	Allows a variety of individual reasons to be considered	Highly demanding to implement, especially in large groups with weak ties
Utilitarianism[22]	Right action produces the most good	Convenient to the majority	Optimises the number of participants	Systematically disadvantages minority populations
Egalitarianism[23]	Favours equality of some sort	Inconvenience shared	Provides equal convenience and opportunity to participate	Insufficient to provide equity to disadvantaged groups
Prioritarianism[24]	Well-being across all individuals, with extra weight given to worse-off individuals	Priority to most affected, most hard-pressed, or least represented	Offers equity for disadvantaged groups	May yield fewer participants overall

For peer review only

REFERENCES

1. Koplan JP, Bond TC, Merson MH, et al. Towards a common definition of global health. *Lancet* 2009;373:1993-5.

2. World Health Organisation. Coronavirus disease (COVID-19) advice for the public: World Health Organisation; 2021 [Available from: https://www.who.int/emergencies/diseases/novel-coronavirus-2019/advice-for-public accessed 17 June 2021.

3. Denstadli JM, Julsrud TE, Hjorthol RJ. Videoconferencing as a mode of communication: a comparative study of the use of videoconferencing and face-to-face meetings. *J Bus Tech Commun* 2012;26:65-91.

4. Amankwah-Amoah J. Stepping up and stepping out of COVID-19: new challenges for environmental sustainability policies in the global airline industry. *J Cleaner Production* 2020;271:123000.

5. International Conference held at Washington for the purpose of fixing a prime meridian and universal day October 1884. Protocols of the proceedings. Washington, DC, 1884.

6. Kurunmäki J, Marjanen J. A rhetorical view of isms: an introduction. *J Political Ideol* 2018;23:241-55.

7. Cummings JN. Geography is alive and well in virtual teams. *Commun ACM* 2011;54:24-6.

8. Aschoff J. Circadian rhythms in man. *Science* 1965;148:1427-32.

9. Morrison-Smith S, Ruiz J. Challenges and barriers in virtual teams: a literature review. *SN Appl Sci* 2020;2:1096.

10. IARC Monographs Vol 124 group. Carcinogenicity of night shift work. *Lancet Oncol* 2019;20:1058-9.

For peer review only

11. Abimbola S, Pai M. Will global health survive its decolonisation? *Lancet* 2020;396:1627-8.

12. Khan M, Abimbola S, Aloudat T, et al. Decolonising global health in 2021: a roadmap to move from rhetoric to reform. *BMJ Glob Health* 2021;6:e005604.

13. Teucher A. LUTZ: look up time zones of point coordinates. R package 0.3.1. 2019. https://CRAN.R-project.org/package=lutz (accessed 5 July 2021).

14. R Development Core Team. R: A language and environment for statistical computing. *R Foundation for Statistical Computing* 2019. https://www.R-project.org/ (accessed 5 July 2021).

15. Center for International Earth Science Information Network - CIESIN - Columbia University. Gridded population of the world, version 4 (GPWv4): population count adjusted to match 2015 revision of United Nations World Population Prospects country totals, revision 11. 2018. https://doi.org/10.7927/H4PN93PB (accessed 5 July 2021).

16. United Nations Department of Economic and Social Affairs Population Division. World population prospects 2019: volume I: comprehensive tables, 2019.

17. United Nations Department of Economic and Social Affairs Population Division. World population prospects 2019: volume II: demographic profiles, 2019.

18. United Nations Development Program. Human development reports: human development data center 2020 [Available from: http://hdr.undp.org/en/data accessed 5 July 2021.

19. Institute for Health Metrics and Evaluation. Global health data exchange Seattle, WA: IHME, University of Washington,; 2021 [Available from: http://ghdx.healthdata.org accessed 22 June 2021.

20. Sidgwick H. The methods of ethics. London: Macmillan 1874.

For peer review only

21. Scanlon TM. What we owe each other. Cambridge, MA: Hardvard University Press 1982.

22. Bentham J. An introduction to the principles of morals and legislation. Oxford: Clarendon Press, 1789.

23. Arneson R. Egalitarianism. In: Zalta EN, ed. The Stanford Encyclopedia of Philosophy. Stanford, CA, 2013.

24. Parfit D. Equality and priority. *Ratio* 2002;10:202-21.

25. Lees N. The Brandt Line after forty years: The more North–South relations change, the more they stay the same? *Rev Int Stud* 2021;47:85-106.

26. Braveman P, Gruskin S. Defining equity in health. *J Epidemiol Community Health* 2003;57:254-8.

27. Robson G, Gibson N, Thompson A, et al. Global health ethics: critical reflections on the contours of an emerging field, 1977-2015. *BMC Med Ethics* 2019;20:53.

For peer review only

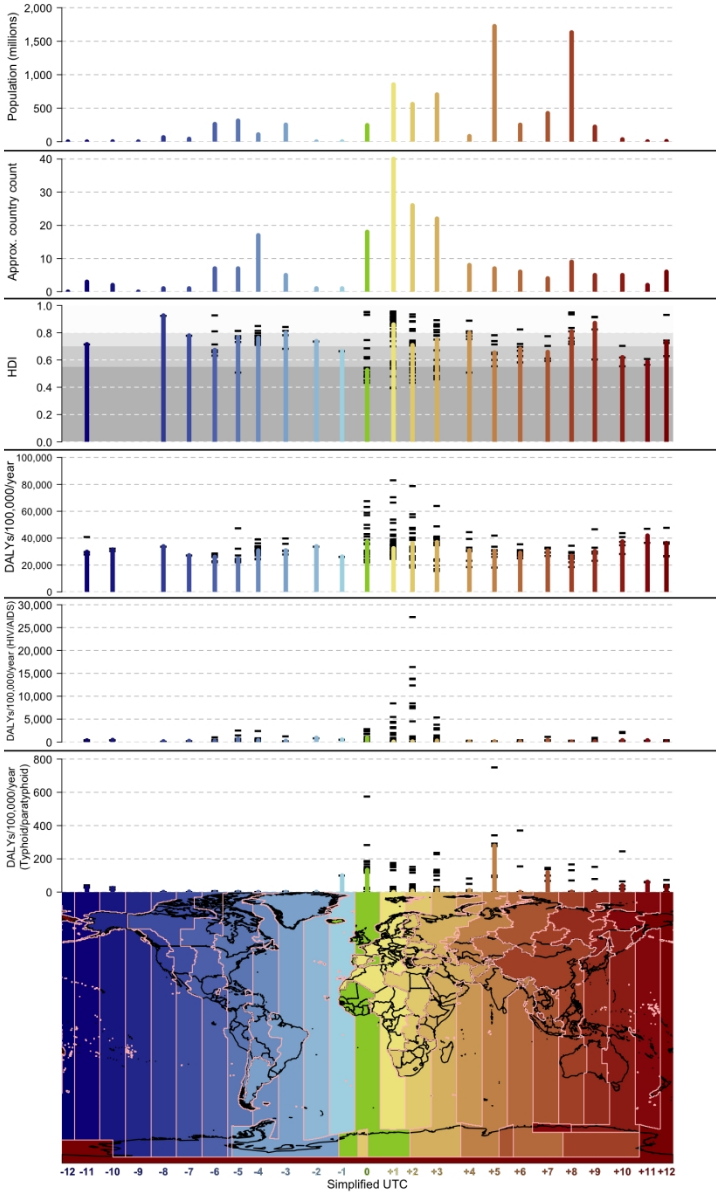

Figure. Global population, countries, human development index, total and disease-specific disability adjusted life years per 100,000 by simplified Coordinated Universal Time offset, 2019-20

518x873mm (72 x 72 DPI)

Supplementary Figure. Static image of interactive three-dimensional UTC split-plot of global population, countries, human development index, total and disease-specific disability adjusted life years per 100,000 by simplified Coordinated Universal Time offset, 2019-20

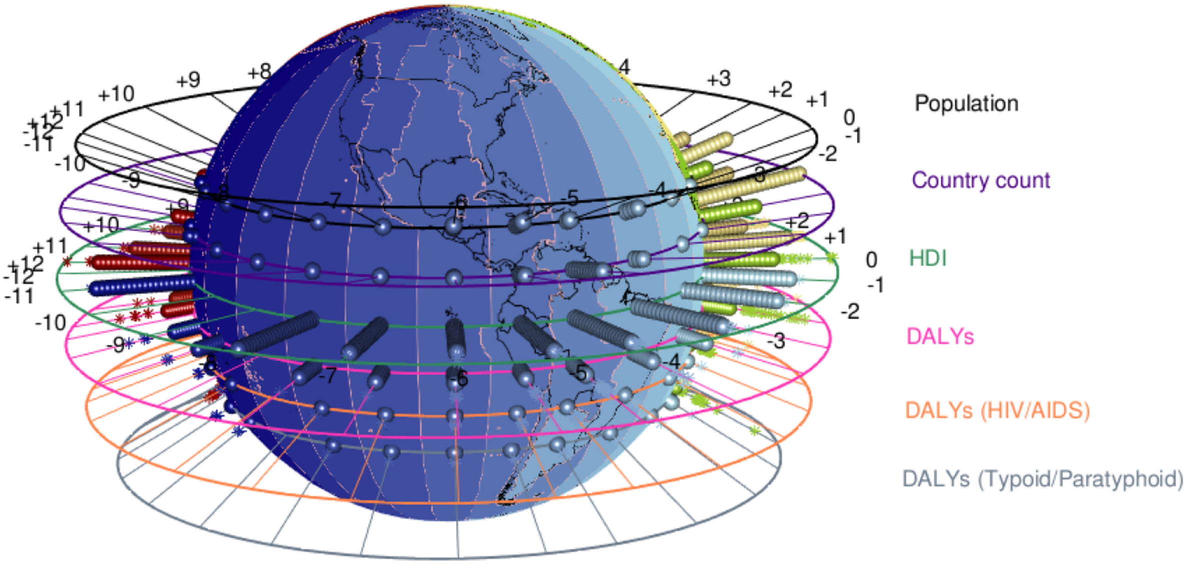

HDI: Human development index; DALYs: disability-adjusted life years; UTC: Coordinated Universal Time.
Interactive version available at:
https://www.stats.otago.ac.nz/~tdavies/utcsplit_health.html
(allow up to 30 seconds to load in browser)

For peer review only

STROBE Statement—checklist of items that should be included in reports of observational studies

	Item No	Recommendation	Page No
**Title and abstract**	1	(*a*) Indicate the study's design with a commonly used term in the title or the abstract	1
		(*b*) Provide in the abstract an informative and balanced summary of what was done and what was found	2-3
**Introduction**			
Background/rationale	2	Explain the scientific background and rationale for the investigation being reported	4-6
Objectives	3	State specific objectives, including any prespecified hypotheses	5-6
**Methods**			
Study design	4	Present key elements of study design early in the paper	6-9
Setting	5	Describe the setting, locations, and relevant dates, including periods of recruitment, exposure, follow-up, and data collection	6-9
Participants	6	(*a*) *Cohort study*—Give the eligibility criteria, and the sources and methods of selection of participants. Describe methods of follow-up<br>*Case-control study*—Give the eligibility criteria, and the sources and methods of case ascertainment and control selection. Give the rationale for the choice of cases and controls<br>*Cross-sectional study*—Give the eligibility criteria, and the sources and methods of selection of participants	6-9
		(*b*) *Cohort study*—For matched studies, give matching criteria and number of exposed and unexposed<br>*Case-control study*—For matched studies, give matching criteria and the number of controls per case	
Variables	7	Clearly define all outcomes, exposures, predictors, potential confounders, and effect modifiers. Give diagnostic criteria, if applicable	6-9
Data sources/ measurement	8*	For each variable of interest, give sources of data and details of methods of assessment (measurement). Describe comparability of assessment methods if there is more than one group	6-9
Bias	9	Describe any efforts to address potential sources of bias	6-9
Study size	10	Explain how the study size was arrived at	6-9
Quantitative variables	11	Explain how quantitative variables were handled in the analyses. If applicable, describe which groupings were chosen and why	6-9
Statistical methods	12	(*a*) Describe all statistical methods, including those used to control for confounding	6-9
		(*b*) Describe any methods used to examine subgroups and interactions	6-9
		(*c*) Explain how missing data were addressed	6-9
		(*d*) *Cohort study*—If applicable, explain how loss to follow-up was addressed<br>*Case-control study*—If applicable, explain how matching of cases and controls was addressed<br>*Cross-sectional study*—If applicable, describe analytical methods taking account of sampling strategy	6-9
		(*e*) Describe any sensitivity analyses	6-9

Continued on next page

For peer review only - http://bmjopen.bmj.com/site/about/guidelines.xhtml

**Results**			
Participants	13*	(a) Report numbers of individuals at each stage of study—eg numbers potentially eligible, examined for eligibility, confirmed eligible, included in the study, completing follow-up, and analysed	9-11
		(b) Give reasons for non-participation at each stage	NA
		(c) Consider use of a flow diagram	NA
Descriptive data	14*	(a) Give characteristics of study participants (eg demographic, clinical, social) and information on exposures and potential confounders	9-11
		(b) Indicate number of participants with missing data for each variable of interest	9-11
		(c) *Cohort study*—Summarise follow-up time (eg, average and total amount)	NA
Outcome data	15*	*Cohort study*—Report numbers of outcome events or summary measures over time	NA
		Case-control study—Report numbers in each exposure category, or summary measures of exposure	NA
		Cross-sectional study—Report numbers of outcome events or summary measures	9-11
Main results	16	(*a*) Give unadjusted estimates and, if applicable, confounder-adjusted estimates and their precision (eg, 95% confidence interval). Make clear which confounders were adjusted for and why they were included	NA
		(*b*) Report category boundaries when continuous variables were categorized	9-11
		(*c*) If relevant, consider translating estimates of relative risk into absolute risk for a meaningful time period	NA
Other analyses	17	Report other analyses done—eg analyses of subgroups and interactions, and sensitivity analyses	9-11
**Discussion**			
Key results	18	Summarise key results with reference to study objectives	11
Limitations	19	Discuss limitations of the study, taking into account sources of potential bias or imprecision. Discuss both direction and magnitude of any potential bias	11-12
Interpretation	20	Give a cautious overall interpretation of results considering objectives, limitations, multiplicity of analyses, results from similar studies, and other relevant evidence	12-15
Generalisability	21	Discuss the generalisability (external validity) of the study results	15
**Other information**			
Funding	22	Give the source of funding and the role of the funders for the present study and, if applicable, for the original study on which the present article is based	17

*Give information separately for cases and controls in case-control studies and, if applicable, for exposed and unexposed groups in cohort and cross-sectional studies.

Note: An Explanation and Elaboration article discusses each checklist item and gives methodological background and published examples of transparent reporting. The STROBE checklist is best used in conjunction with this article (freely available on the Web sites of PLoS Medicine at http://www.plosmedicine.org/, Annals of Internal Medicine at http://www.annals.org/, and Epidemiology at http://www.epidem.com/). Information on the STROBE Initiative is available at www.strobe-statement.org.

For peer review only

For peer review only - http://bmjopen.bmj.com/site/about/guidelines.xhtml
